# The First Molecular Detection of *Theileria luwenshuni* from *Haemaphysalis mageshimaensis* on Orchid Island, Taiwan, with No Evidence of SFTSV

**DOI:** 10.3390/pathogens14030241

**Published:** 2025-03-03

**Authors:** Pai-Shan Chiang, I-Jung Tsai, Yuan-Wei Hu, Hung-Jui Chen, I-Jen Chen, Hwa-Jen Teng, Shiu-Ling Chen

**Affiliations:** 1 Center for Diagnostics and Vaccine Development, Centers for Disease Control, Ministry of Health and Welfare, No. 6, Linsen S. Road, Taipei City 10050, Taiwan; 2 Taiwan Forestry Research Institute, No. 53, Nanhai Road, Taipei City 10066, Taiwan

**Keywords:** *Theileria luwenshuni*, *Haemaphysalis mageshimaensis*, tick, Orchid Island, Taiwan

## Abstract

Theileriosis is considered an economically important disease that may decrease productivity and cause a high mortality rate in livestock. Only a few studies have reported *Theileria* spp., such as *T. sergenti* and *T. buffeli*, in recent decades in Taiwan. In the present study, 401 ticks have been collected on Orchid Island in June 2022 and April 2023. Our environmental investigation for SFTSV unintentionally discovered *T. luwenshuni* in *Haemaphysalis mageshimaensis* on Orchid Island via PCR. The PCR products were sequenced, and the detected 18S rRNA gene sequences shared a 99.65–99.93% identity with *T. luwenshuni* sequences from ticks and ruminants in Myanmar and China. Despite the difficulty in clarifying the source of *T. luwenshuni* within neighboring regions, our findings provide an updated distribution of *T. luwenshuni* in Asia. This is not only the first time that *T. luwenshuni* was found in *H. mageshimaensis* but also the first report of *T. luwenshuni* on Orchid Island, Taiwan. Our study indicates that ruminants may be at risk of infection. Therefore, further investigations are needed to determine the distribution of *T. luwenshuni* among ruminants on Orchid Island and in Taiwan.

## 1. Introduction

*Theileria* species infect various types of animals, such as cattle, horses, and sheep [1]. *Theileria* spp. can be divided into transforming and non-transforming groups based on whether schizonts develop in leukocytes. Non-transforming *Theileria* species do not develop schizonts in leukocytes and, therefore, do not induce transformation. Consequently, transforming *Theileria* species can cause fatal lymphoproliferation, while non-transforming *Theileria* species primarily cause anemia [2,3].

Theileriosis refers to the reproduction of *Theileria* spp. in the host and is considered an economically important disease [4]. It threatens the livestock economy by decreasing the productivity and increasing the mortality rate of hosts; for instance, *T. lesteoquardi*, *T. luwenshuni*, and *T. uiolenbergi* cause high mortality rates in small ruminants [5]. Moreover, studies have reported a high prevalence of pathogenic *Theileria* parasites in China and Pakistan, which has been associated with economic damage, particularly in the cattle industry [6,7]. Theileriosis has been reported in hosts of specific species. For instance, *T. parva* and *T. annulata* were found in cattle, *T. equi* in horses, and *T. lestoquardi* and *T. luwenshuni* in sheep and goats. Despite some evidence from Ghana indicating that *Theileria* spp. may be less likely to infect humans, further investigation is required to clarify the human–vector interactions of *Theileria* spp. [8]. Only a few studies have reported information on *Theileria* spp. in recent decades in Taiwan. Wang et al. conducted a survey of *Theileria* spp. infection in cattle and found piroplasms in 4 of 105 blood samples. In addition, they reported the infection of *T. sergenti* and *T. buffeli* by allele-specific PCR in 15 of 105 blood samples in 1998 [9]. Another survey executed by Chan et al. in 2010 suggested that equine piroplasmosis was not detected in 489 blood samples from horses [10]. To date, no study has investigated sheep, goats, or ticks in Taiwan.

Ticks and their pathogens may travel across regions and countries with migrating birds [11]. Recently, Yin et al. reported that *T. luwenshuni* [12] was found in other Eurasian countries, including Korea, Türkiye, northern India, and the Mediterranean region [13,14,15], and that certain tick-borne pathogens (i.e., severe fever with thrombocytopenia syndrome virus, *Borrelia burgdorferi,* and *Babesia divergens*) were first discovered or frequently found in cold and/or temperate areas. However, *T. luwenshuni* has recently been reported in Myanmar [16]. These recent reports imply that *T. luwenshuni* and its corresponding vectors are of increasing importance in the tropics.

Severe Fever with Thrombocytopenia Syndrome Virus (SFTSV) has emerged as a significant public health threat across East Asia since its identification in China in 2009 [17]. Since then, it has spread across in China and neighboring countries such as Japan, South Korea, and Vietnam [18,19,20,21]. The virus causes Severe Fever with Thrombocytopenia Syndrome (SFTS), a potentially fatal illness characterized by fever, thrombocytopenia, leukocytopenia, and, in severe cases, multi-organ failure [22]. SFTSV was transmitted primarily by the tick *Haemaphysalis longicornis* but had also been detected in several tick species. Moreover, it has been detected in ruminants, wild animals, and stray animals [23,24]. The presence of SFTSV in both ticks and animals, suggests an increasing transmission risk of SFTSV. In 2019, the first indigenous SFTS case was confirmed in Taiwan [25]. The following investigation was conducted with no findings of SFTSV [26]. In 2022, another SFTS case was confirmed in Orchid Island, Taiwan.

Hence, the purpose of our study was to conduct an environmental investigation due to the confirmation of indigenous SFTS human cases in 2022. Our environmental investigation received a negative result of SFTSV which provides evidence for understanding the prevalence of SFTSV on Orchid Island. The other purpose of our study was to investigate the collected ticks for *Hepatozoon*, *Babesia*, and *Theileria* species with molecular methods.

## 2. Materials and Methods

An environmental investigation was conducted on Orchid Island in June 2022 and April 2023. During the investigation, 401 wild ticks were collected from local vegetation by flagging, which was conducted between June 2022 and April 2023 with two flags per 50 m^2^ over a period of 4 days. All the collected ticks were kept alive at room temperature until they were transferred to the laboratory on dry ice and stored at −80 °C. All the collected ticks were examined under a dissecting microscope (Leica M205C, Wetzlar, Germany) and identified by morphological characteristics using published keys [27]. After morphological identification, tick species identity was confirmed by PCR and sequencing of the 18S and 12S rRNA genes based on representative ticks (Table S1).

The collected samples were analyzed according to the life stage of ticks. Nymphal and adult ticks from each individual tick sample were homogenized separately via the same method. Larval ticks were separated by collected location and pooled into groups of 2–10 ticks, with an initial pool of 10 ticks. If fewer than 10 ticks remained, they were combined into a smaller pool. Each pool was homogenized in a microcentrifuge tube containing 500 µL of phosphate-buffered saline and stainless-steel beads by using a TissueLyser LT (Qiagen Benelux BV, Venlo, The Netherlands). The supernatants of the homogenized mixtures were collected and stored at −80 °C until subsequent use. To determine whether the collected ticks were infected with SFTSV and tick-borne pathogens recently reported in Asia, molecular tests were performed.

In brief, pathogen RNA was isolated from 140 µL of a homogeneous tick mixture by using the QIAamp viral RNA mini kit (Qiagen GmbH, Hilden, Germany) and then reverse-transcribed (SuperScript IV reverse transcriptase, Invitrogen, Waltham, MA, USA); SFTSV-specific fragments were then detected via PCR (SapphireAmp fast PCR kit, TaKaRa Bio, Kusatsu, Japan) with specific primers (Table S1). In addition, nucleic acids extracted from homogeneous tick mixtures were tested without reverse transcription for piroplasm parasites using a primer set that can simultaneously detect 18S rRNA from *Babesia*, *Theileria*, and *Hepatozoon* species (Table S1). The genomes of tick-borne pathogens (SFTSV and *B. microti*) and sterile water were included as positive and negative/blank controls, respectively, in each test round. The PCR amplicons used for tick species identification and detection of tick-borne pathogens were further sequenced using an ABI 3730 XL DNA Analyzer (Applied Biosystem, Inc., Foster City, CA, USA). The nucleotide sequences acquired in this study were compared against the NCBI GenBank database by BLAST (https://blast.ncbi.nlm.nih.gov/Blast.cgi) search to identify the species. In addition, the acquired sequences were aligned with sequences already registered in the GenBank database using CLUSTALW. Molecular phylogenetic trees of ticks and tick-borne pathogens were reconstructed by the neighbor-joining method with the Kimura two-parameter model and 1000 bootstrap resampling replicates, as implemented in MEGA software version 11.0.11. Pairwise deletion was used for gaps/missing data.

## 3. Results

**Figure 1:**
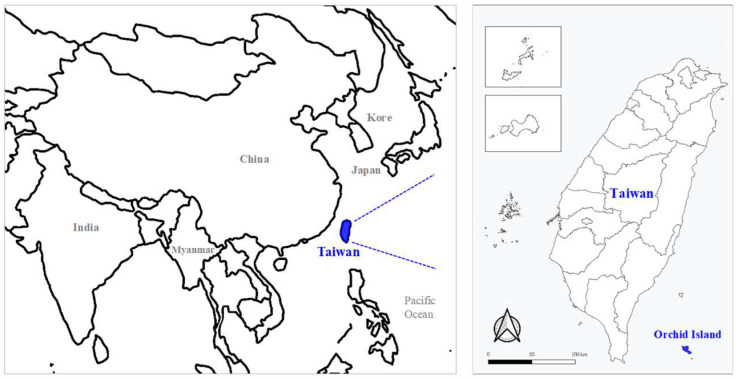
Map showing the relative location and the size of Orchid Island in East Asia.

The indigenous SFTS human cases were confirmed on Orchid Island (Figure 1) in 2022. Thus, the environmental investigation on Orchid Island was carried out to identify prevalence of SFTSV in the environment (Figure 1) in June 2022 and April 2023.

**Figure 2:**
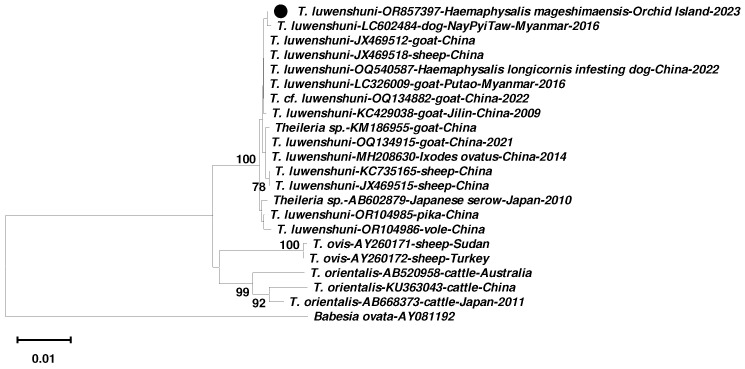
**Phylogenetic relationships of the sequence of the *T. luwenshuni* strain.** A neighbor-joining phylogenetic tree was constructed based on partial 18S rRNA gene sequences (1439 nt long) of *Theileria* spp. Bootstrap values (>70%) at the nodes of the tree are shown as percentages derived from 1000 samplings. The scale bar denotes the number of nucleotide substitutions per site along the branches. The solid dot indicates the *T. luwenshuni* strain detected in this study.

A total of 401 *H. mageshimaensis* samples were captured by flagging them from vegetation, and subsequently identified with characteristics—for instance, the flat anterior surface of the basis capitulum perpendicular to the longitudinal axis of the hypostome. The samples included 377 larvae, 23 nymphs, and 1 adult; and 40 pooled larvae samples, 23 nymphs, and 1 adult were analyzed (Table S2). Taxonomic identification was based on 18S and 12S rRNA gene sequences, and the accession numbers for the representative ticks are shown in Figure S1. Among 64 samples, we received a negative result for SFTSV in all samples and found a positive result for the 18S rRNA gene in a nymph sample. The PCR product was subjected to DNA sequencing analysis. The positive nymphal *H. mageshimaensis* sample was collected in April 2023 from a grassland inhabited by wild goats. The 18S rRNA gene sequence was identified as a *Theileria* parasite and phylogenetically grouped within a cluster of *T. luwenshuni,* which is distinct from other *Theileria* spp. clusters (Figure 2).

The 18S rRNA gene sequence of *T. luwenshuni* that we detected on Orchid Island (OR857397) showed a high nucleotide sequence identity with *T. luwenshuni* sequences from ticks, ruminants, and wild animals in Myanmar and China. These included sequences with GenBank accession numbers LC326009 and LC602484 from Myanmar and OQ540587, OQ134882, JX469518, KC429038, MH208630, OR104985, and OR104986 from China (Table S3).

## 4. Discussion

Severe Fever with Thrombocytopenia Syndrome Virus is a tick-borne virus that leads to a 30% morality rate. It was first identified in China in 2009. In Taiwan, it was first reported in humans in 2019 [25]. Studies have shown the SFTSV RNA was detected in 29.9% of ruminants and 23% of dogs and cats in Taiwan [23]. However, no SFTSV RNA has been reported from animals or ticks on Orchid Island. In 2022, indigenous SFTS human cases were confirmed on Orchid Island. Thus, an environmental investigation has been conducted to clarify the potential infective source. Despite the prevalence of SFTSV in Taiwan being around 23–29.9% in animals, no SFTSV has been detected in our 401 ticks collected from Orchid Island. The samples we collected from Orchid Island were further examined by the nested-PCR to examine if it was infected with *Theileria* spp., *Babesia* spp., and *Hepatozoon* spp. We discovered that a nymphal *H. mageshimaensis* carried the DNA of *Theileria luwenshuni*, which has not been reported before on Orchid Island. The sequence of our finding shares a high similarity with LC326009 and LC602484 from Myanmar and OQ540587, OQ134882, and JX469518 from China. Furthermore, a phylogenetic analysis revealed that our *Theileria* sequences were grouped within the *T. luwenshuni* clade, with a high sequence similarity (>99%) to previously reported isolates from Myanmar and China.

Bovine theileriosis has existed in Tibet and China for more than 100 years [28]. Orchid Island is geographically close to many provinces or regions in which *Theileria* parasites have been detected, such as Korea, Türkiye, northern India, and the Mediterranean region [13,14,15]. These records indicate that the presence of *Theileria* parasites on the islet may result from bovine theileriosis on the Asian continent and on neighboring islands. Although reports of *T. luwenshuni*-infected birds are still limited, the distribution of *T. luwenshuni* may be influenced by the movement of East Asian migratory birds across countries [29]. However, reports on the economic importance of *T. luwenshuni* are limited. A study by Philip et al. revealed that *T. luwenshuni* was associated with disease and mortality during an outbreak in sheep in approximately 2005 in Great Britain [30]. In contrast, an outbreak of *T. luwenshuni*-induced theileriosis in sheep in India did not result in any reported illness [4]. Together, these studies indicate that *T. luwenshuni* may lead to illness and mortality.

Initially, *T. luwenshuni* was reported to cause theileriosis in small ruminants, such as cattle, sheep, and goats [31]. A recent study highlighted the ability of *T. luwenshuni* to infect mice, revealing the complexity of its transmission cycle [32]. Furthermore, we identified *T. luwenshuni* in a new putative vector, *H. mageshimaensis,* which has been found on Artiodactyla animals, including goats, cattle, buffaloes, and pigs, on Orchid Island [33]. However, further investigations are needed to clarify the transmission cycle and the impact of *T. luwenshuni* on ruminants.

In conclusion, this is not only the first time that *T. luwenshuni* has been found in *H. mageshimaensis* but also the first report of *T. luwenshuni* on Orchid Island, Taiwan. Our study indicates that ruminants may be at risk of infection. However, further epidemiological studies are necessary in order to reveal the infection status of *T. luwenshuni* in domestic goats and other livestock on Orchid Island and in Taiwan. More importantly, our results provide an insight into the distribution of tick-borne parasites in Asia. The limitation of this study is that the primers that we used to screen for *Theileria* spp. may also detect *Babesia* spp. and *Hepatozoon* spp. Thus, we are not able to exclude the possibility of co-infection. Nevertheless, the limitation does not compromise the finding of *T. luwenshuni*.

## Data Availability

The original contributions presented in this study are included in the article/Supplementary Materials. Further inquiries can be directed to the corresponding author.

## Supplementary Materials

The following supporting information can be downloaded at: https://www.mdpi.com/article/10.3390/pathogens14030241/s1, Figure S1A: Phylogenetic relationships based on 16S rRNA gene sequences of ticks collected in this study and reference sequences. Figure S1B: Phylogenetic relationships based on 12S rRNA gene sequences of ticks collected in this study and reference sequences. Table S1: Oligonucleotide primers used in this study [34,35,36,37]. Table S2: The pooling information. Table S3: Percentage identities of partial 18S rRNA gene sequences of *T. luwenshuni* (1439 bp) from Orchid Island, Myanmar and China.
