# Supplementary material for: The First Molecular Detection of Theileria luwenshuni from Haemaphysalis mageshimaensis on Orchid Island, Taiwan, with No Evidence of SFTSV
Source: Pathogens. 2025 Mar 3;14(3):241. doi: 10.3390/pathogens14030241 (PMC11945472; doi:10.3390/pathogens14030241)
Supplement: Supplementary file 1 [file pathogens-14-00241-s001.zip › Figure S1.pdf]

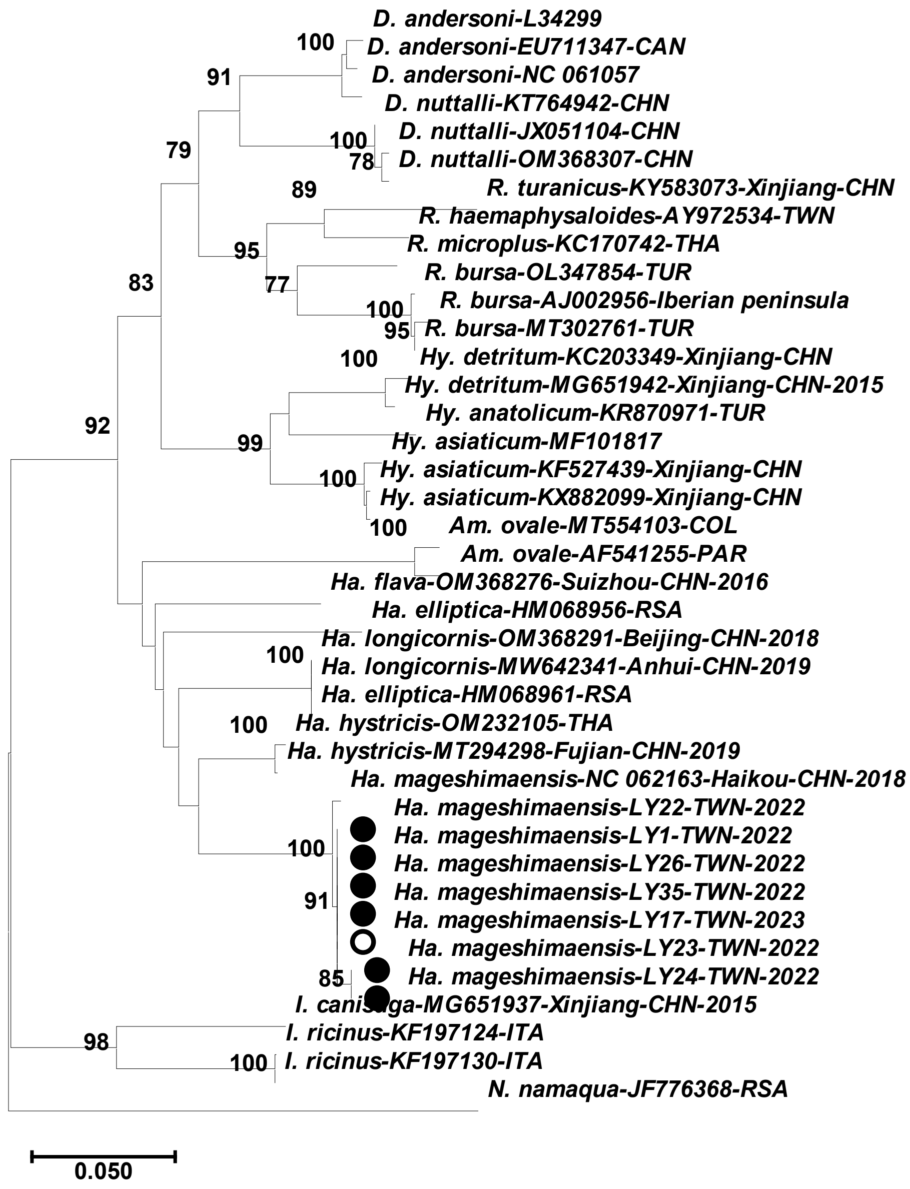

Figure S1A Phylogenetic relationships based on 16S rRNA gene sequences of ticks collected in this study and reference sequences. Bootstrap values (>70%) are shown at the nodes of the neighbor-joining phylogenetic tree as percentages derived from 1,000 samplings. The scale bar denotes the number of nucleotide substitutions per site along the branches. The open circle and solid dots indicate the tick with *T. luwenshuni* and other ticks collected in this study, respectively. The analyzed 16S rRNA nucleotide sequences from the ticks were approximately 450-nt long, ranging from position 6,982 to position 7,404, based on the complete mitochondrial sequence of *D. andersoni* (NC_061057). Abbreviations: CAN, Canada; CHN, China; COL, Colombia; ITA, Italy; PAR, Paraguay; RSA, Republic of South Africa; THA, Thailand; TUR, Turkey; TWN, Taiwan. *Am.*, *Amblyomma*; *D.*, *Dermacentor*; *Ha.*, *Haemaphysalis*; *Hy.*, *Hyalomma*; *R.*, *Rhipicephalus*; *I.*, *Ixodes*; *N.*, *Nuttalliella*.

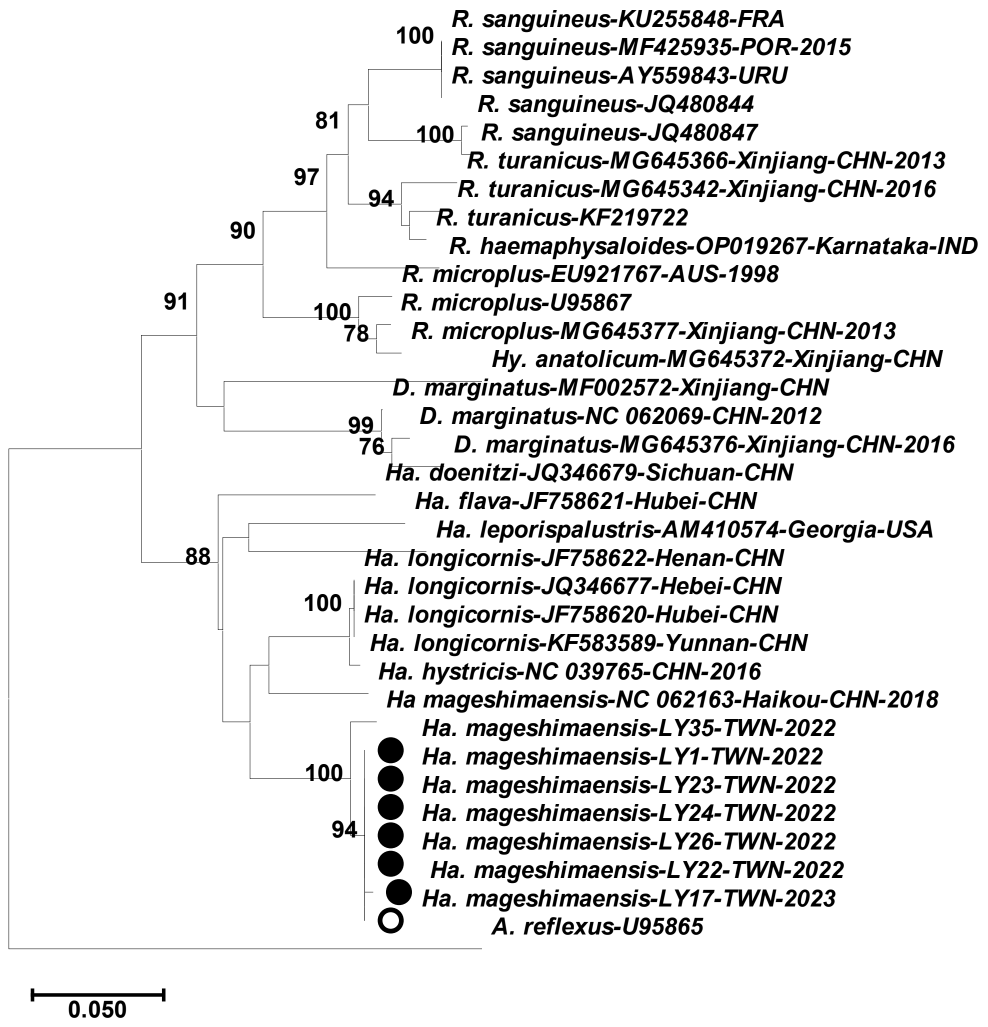

Figure S1B Phylogenetic relationships based on 12S rRNA gene sequences of ticks collected in this study and reference sequences. Bootstrap values (>70%) are shown at the nodes of the neighbor-joining phylogenetic tree as percentages derived from 1,000 samplings. The scale bar denotes the number of nucleotide substitutions per site along the branches. The open circle and solid dots indicate the tick with *T. luwenshuni* and other ticks collected in this study, respectively. The analyzed 12S rRNA nucleotide sequences of the ticks were approximately 350-nt long, ranging from position 8,141 to position 8,478, based on the complete mitochondrial sequence of *H. hystricis* (NC_039765). Abbreviations: AUS, Australia; CHN, China; FRA, France; IND, India; POR, Portugal; TWN, Taiwan; URU, Uruguay; USA, United States of America. *A.*, *Argas*; *D.*, *Dermacentor*; *Ha.*, *Haemaphysalis*; *R.*, *Rhipicephalus*.
