# Supplementary material for: The First Molecular Detection of Theileria luwenshuni from Haemaphysalis mageshimaensis on Orchid Island, Taiwan, with No Evidence of SFTSV
Source: Pathogens. 2025 Mar 3;14(3):241. doi: 10.3390/pathogens14030241 (PMC11945472; doi:10.3390/pathogens14030241)
Supplement: Supplementary file 1 [file pathogens-14-00241-s001.zip › Table S1.pdf]

Table S1. Oligonucleotide primers used in this study

Primer	Sequence (5′–3′)	Target size/Targeted gene	Reference
**(A) Tick species identification**			
T1B	5’-AAACTAGGATAGATACCCT-3’	360 bp/12S rRNA	[34]
T2A	5’-AATGAGAGCGACGGGCGATGT-3’		
16S+1	5’-CTGCTCAATGATTTTTTAAATTGCTGTGG-3’	460 bp/16S rRNA	[35]
16S-1	5’-CCGGTCTGAACTCAGATCAAGTA-3’		
**(B) *Theileria, Babesia, and Hepatozoon* parasite detection and identification**			
BTH 18S 1st F	5’-ACGGCTACCACATCTAAGGAAGGC-3’	1st PCR	[36]
BTH 18S 1st R	5’-TCTCTCAAGGTGCTGAAGGA-3’		
BTH 18S 2nd F	5’-GGCTCATTACAACAGTTATAGTTTATTTG-3’	1.4–1.6 kb/18S rRNA for *Theileria* identification and sequencing	
BTH 18S 2nd R	5’-CGGTCCGAATAATTCACCGGAT-3’		
Inner Seq	5’-AAGTCTGGTGCCAGCAGC-3’		
**(C) SFTSV detection**			
NP-2F (1st PCR)	5’ -CATCATTGTCTTTGCCCTGA-3’	461 bp for primary PCR, 346 bp for nested PCR/S segment	[37]
NP-2R (1st PCR)	5’-AGAAGACAGAGTTCACAGCA-3’		
N2F (2nd PCR)	5’ -AAYAAGATCGTCAAG GCATCA-3’		
N2R (2nd PCR)	5’-TAGTCTTGGTGAAGGCATCTT-3’		

1st PCR: primary PCR; 2nd PCR: nested PCR
