# Supplementary material for: The First Molecular Detection of Theileria luwenshuni from Haemaphysalis mageshimaensis on Orchid Island, Taiwan, with No Evidence of SFTSV
Source: Pathogens. 2025 Mar 3;14(3):241. doi: 10.3390/pathogens14030241 (PMC11945472; doi:10.3390/pathogens14030241)
Supplement: Supplementary file 1 [file pathogens-14-00241-s001.zip › Table S3.pdf]

Table S3. Percentage identities of partial 18S rRNA gene sequences of *T. luwenshuni* (1,439 bp) from Orchid Island, Myanmar and China.

Locations	18S rRNA gene sequence (accession number/host or vector)	(1)	(2)	(3)	(4)	(5)	(6)	(7)	(8)	(9)
Orchid Island	(1) OR857397/*H. mageshimaensis*	–	–	–	–	–	–	–	–	–
Myanmar	(2) LC326009/goat	99.93%	–	–	–	–	–	–	–	–
	(3) LC602484/dog	99.93%	99.86%	–	–	–	–	–	–	–
China	(4) OQ540587/*H. longicornis*-infested dog	99.93%	100.00%	99.86%	–	–	–	–	–	–
	(5) OQ134882/goat	99.93%	100.00%	99.86%	100.00%	–	–	–	–	–
	(6) JX469518/sheep	99.93%	100.00%	99.86%	100.00%	100.00%	–	–	–	–
	(7) KC429038/goat	99.86%	99.93%	99.79%	99.93%	99.93%	99.93%	–	–	–
	(8) MH208630/*I. ovatus*	99.86%	99.93%	99.79%	99.93%	99.93%	99.93%	99.86%	–	–
	(9) OR104985/pika	99.79%	99.86%	99.72%	99.86%	99.86%	99.86%	99.79%	99.79%	–
	(10) OR104986/vole	99.65%	99.72%	99.58%	99.72%	99.72%	99.72%	99.65%	99.65%	99.86%
